# Induced Genetic Variations in Fruit Trees Using New Breeding Tools: Food Security and Climate Resilience

**DOI:** 10.3390/plants10071347

**Published:** 2021-07-01

**Authors:** Muhammad Naeem Sattar, Zafar Iqbal, Jameel M. Al-Khayri, S. Mohan Jain

**Affiliations:** 1 Central Laboratories, King Faisal University, Al-Ahsa 31982, Saudi Arabia; mnsattar@kfu.edu.sa (M.N.S.); zafar@kfu.edu.sa (Z.I.); 2 Department of Agricultural Biotechnology, College of Agriculture and Food Sciences, King Faisal University, Al-Ahsa 31982, Saudi Arabia; 3 Department of Agricultural Sciences, PL-27, University of Helsinki, 00014 Helsinki, Finland; mohan.jain@helsinki.fi

**Keywords:** mutagenesis, TILLING, genome editing, targeted mutation, CRISPR-Cas, transgene-free, fruit trees

## Abstract

Fruit trees provide essential nutrients to humans by contributing to major agricultural outputs and economic growth globally. However, major constraints to sustainable agricultural productivity are the uncontrolled proliferation of the population, and biotic and abiotic stresses. Tree mutation breeding has been substantially improved using different physical and chemical mutagens. Nonetheless, tree plant breeding has certain crucial bottlenecks including a long life cycle, ploidy level, occurrence of sequence polymorphisms, nature of parthenocarpic fruit development and linkage. Genetic engineering of trees has focused on boosting quality traits such as productivity, wood quality, and resistance to biotic and abiotic stresses. Recent technological advances in genome editing provide a unique opportunity for the genetic improvement of woody plants. This review examines application of the CRISPR-Cas system to reduce disease susceptibility, alter plant architecture, enhance fruit quality, and improve yields. Examples are discussed of the contemporary CRISPR-Cas system to engineer easily scorable PDS genes, modify lignin, and to alter the flowering onset, fertility, tree architecture and certain biotic stresses.

## 1. Introduction

Conventional breeding has been the sole source of genetic improvement in fruit crops for various traits. Classical approaches to introduce a promising trait in an elite cultivar require the introgression of related alleles through multiple generations of selection. For example, the introduction of a disease-resistance trait into a high yielding cultivar is commenced by crossing it with a disease-resistant cultivar, followed by recurrent backcrossing with the elite parent to sustain the genetic potential of the elite cultivar besides conserving the newly introduced resistance allele. Usually, the whole process encompasses several generations to stabilize the resistance allele in the elite background. Fruit crop breeding have certain limitations, which may include outcross reproduction, prolonged juvenility, and enormous genome landscapes [1,2]; therefore, it requires decades to improve such traits. The obligate outcrossing nature of fruit trees amalgamates classical breeding for genotypic and phenotypic traits. A relevant example to illustrate this dilemma is the development of resistance to apple scab. Hough et al. [3] conducted a wide range of crosses between an elite apple cultivar and a genetically compatible wild-type cultivar as the source of resistance to apple scab. However, over several decades of continuous breeding, the resultant cultivars lost the fruit quality traits [4]. The application of marker-assisted selection, such as marker-assisted breeding (MAB), marker-assisted selection (MAS) and genome-wide association mapping (GWAS) for quantitative trait loci (QTLs), may contribute to shorten the selection process, but not bypass the generations of backcrossing [5]. For example, apples, cucumbers, mandarins, peaches, and strawberries have been substantially improved [6,7,8]. Fast-track breeding approaches may possibly overcome extreme juvenility in fruit trees via the transgenic expression of the desired genes. The breeding time for fruit trees can be shortened to one-fifth of the conventional crossbreeding approaches [9]. For example, the apple cultivar ‘Pinova’ was transformed to impart early flowering by expressing a MADS-box gene from *Betula pendula* [10]. In another study, null segregants of fire blight and apple scab resistant apples were generated within seven years [11]. Similarly, Endo et al. [7] successfully substituted the genetic background of mandarin through an integrated transgenic approach and MAS to transfer CTV resistance from a transgenic trifoliate orange. However, to obtain the null segregants, the transgene should be segregated out from the elite parental background through backcrossing with the recurrent parent. The detachability of the T-DNA transgene can be confirmed through comparative genomic hybridization (CGG) and next-generation sequencing (NGS) approaches [9]. In fast-track breeding, MAS plays a critical role to increase the selection efficiency in the backcrossed progenies.

**Figure 1:**
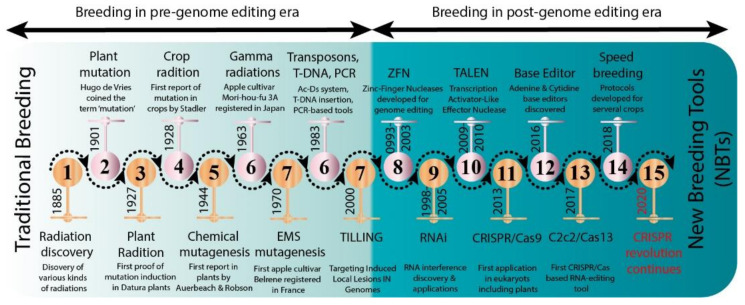
Historic timeline for mutagenesis in plants.

**Figure 2:**
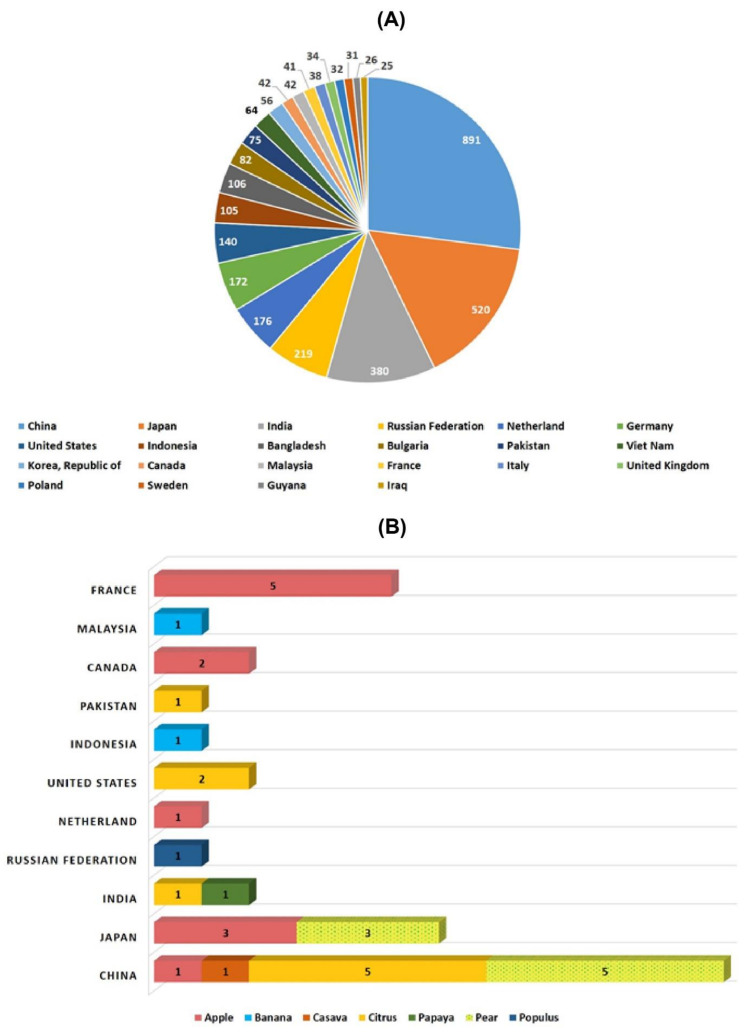
Number of mutant varieties released in top 22 countries (**A**) and number of mutant tree varieties of assorted fruit species in selected countries (**B**).

Several new strategies were introduced in the middle of the 20th century to enrich the genetic diversity of fruit trees. Mutagenesis has been used to facilitate plant breeding since the 1920s with the discovery that mutations induced with physical (gamma irradiation) or chemical mutagen treatment can be inherited [12]. Importantly, with the discovery of X-rays, a subsequent series of induced mutations were conceptualized in plants and animals (Figure 1). In 1934, the first commercial mutant tobacco variety was produced [13]; since then, mutant crop cultivars have been continuously registered globally (Figure 2A,B). It was not until 1963 that the first mutated apple cultivar “Mori-hou-fu 3A” was developed in Japan through gamma rays. The following year, a sweet cherry (*Prunus avium* L.) cultivar “Compact Lambert” was developed in Canada (Table 1). The use of chemical mutagen “EMS” was successfully applied for mutating apples to develop another mutant cultivar “Belrene” in France in 1970. The application of physical mutagens was also successful in tree breeding. For example, grapefruit (*Citrus* × *paradisi*) cultivar “Rio Red” and clementia (*Citrus celementina*) cultivar “Nero” were developed using thermal neutrons and fast neutrons in 1970 and 2006 in USA and Spain, respectively. Various hybridization methods were also developed to produce hybrids between sexually incompatible species by disrupting the meiotic cell division to form polyploids, followed by the restoration of meiosis. Additionally, hybridization approaches also included chromosomal additions/subtractions or the fusion of protoplasts from sexually incompatible species [14]. The genetic background of the elite crop cultivars was further broadened through chemical or physical mutagenesis to increase the desirable alleles in the elite lines.

The advent of plant transformation approaches including *Agrobacterium*-mediated, particle bombardment or electroporation-mediated and chemical transfections through protoplasts allowed the development of transgenic plants with the specific genetic constructs from any biological source. Although these methods involve in vitro culturing, many fruit tree species are recalcitrant to transformation and regeneration. However, fruit crops amenable to in vitro culturing were successfully transformed to directly introgress new genes without recurrent backcrossing [15]. However, the resulting transgenic plants faced regulatory complexities and gave rise to a dichotomy between the product and process regulatory framework [16]. These legalities have some practical implications because even if the gene transfer is intraspecific (involving plants from the same species), it still left some cargo of a transgene such as the remnants of the genetic markers or parts from the bacterial plasmid itself or the T-DNA of *A. tumefaciens* [17].

Genome editing (GE) technology has revolutionized fruit crop breeding [18]. GE encompasses three types of specific site-directed nucleases (SNDs)—i.e., SDN-1, SDN-2, and SDN-3—to cause double-stranded breaks (DSBs) at pre-defined genome targets. The major advantage of SDNs is the targeted DNA cleavage and the subsequent use of cellular machinery to introduce the desired change during the repairing processes. These DSBs are imprecisely repaired by the cell DNA repair mechanism and cause insertion and deletion (indels) mutation over all dysfunctions of the gene of interest without introducing any foreign element into the genome. The entire process is well-regulated and, at the sequence level, the indel mutants cannot be distinguished from natural variations and/or irradiated or chemical mutants [19]. Four SDN-based GE techniques include meganucleases, zing-finger nucleases (ZFNs), transcription activator-like effector nucleases (TALENs), and the most recent clustered regularly interspaced short palindromic repeat/CRISPR-associated proteins (CRISPR/Cas), respectively. These GE techniques are based upon biological molecules with a DNA-binding domain and the cleavage activity (through nucleases). The mutants developed with chemical or physical mutagen treatment are usually hemizygous and their homozygosity is achieved through either filial segregations or recurrent backcrosses to fix the introduced mutations. Nevertheless, this is not a case with site-directed or targeted mutagenesis using GE, and GE shared another edge that is its multiplexing capabilities—i.e., simultaneous targeting of multiple genes or copies of a gene. This characteristic can be extremely useful to target homologous genes in polyploid fruit trees. The recent advancements in GE to substitute a single nucleotide allows individual base swapping in a DNA strand [20]. These developments in GE can help to overcome the GMO regulatory frameworks because it leaves no trace of a transgene or exogenous source in the targeted genome. Moreover, there is evidence that the remnants of *Agrobacterium* T-DNA have a role in the evolution of some plant families [21]. Thus, the boundary between natural and engineered crop species may become more blurred after such evidence and ultimately gain the attention of the scientific community to revise the regulatory framework, at least for GE crops.

## 2. Tree Breeding under Climate Change

The response of trees to any global climate change scenario is a pressing question for natural vegetation and man-made plantations [22]. Climate change is a major threat to tree plantations due to fluctuations in annual precipitation, drought, heat, salinity, and enhanced insect infestations [23]. Investigations to explore the ability and genetic basis of adaptation to global climate change in ecologically and industrially distinct tree species to cope with abiotic and biotic stresses are key research lines in plant science [24]. However, this knowledge has rarely been translated into conserving the genetic resources or bringing the genetic improvements to woody perennials.

The objective of most tree breeding programs is to gradually improve tree populations through recurrent selection cycles and verifications [25]. Traditionally, tree breeding mostly relies on phenotypically selecting superior candidates from the natural or planted stands. It constitutes the base population and further selection builds a pool of selected population with elite donors. Furthermore, these selected populations are then tested for progeny trials and the reselection of parents [26]. However, such selections may cause genetic erosions in the overall populations due to inbreeding depression. The production of hybrids and subsequent backcrossing may accelerate classical breeding with the aim of harnessing heterotic effects by virtue of dominance or over-dominance, tree adaptability and increased yield [27]. Among other potential applications, hybrid breeding has been widely applied to maximize the tree crown perimeter, tree height, conferring resistance to *Fusarium* spp. [28], and to chestnut blight from wild donor tree plants into American chestnut populations [29].

The most promising alternative approach towards tree breeding is molecular- marker-assisted selection (MAS) and molecular-assisted breeding (MAB) [30]. MAS and MAB tools can be effective in pyramiding simple Mendelian traits regulated by a few genes but have limited utility for selection against complex genetic traits in trees [31]. Moreover, MAS and MAB cannot be effective due to fluctuations in allelic frequencies over generations and therefore cannot explain genetic variations for complex traits [1]. To circumvent these limitations, the use of the genomic selection (GD) approach is suitable rather than phenotypic selection-based traditional breeding using MAS and MAB. Despite having a relatively short history, the technique has been successfully implemented in plant breeding. It can substantially reduce the long breeding cycles for tree breeding and positively enhance the genetic gain over time [31]. In the GS approach, a large number of molecular markers are used to analyze the cumulative effects of QTLs evenly distributed over the genome. Therefore, it makes GS much more efficient for tree breeding due to the possibility to assess the individual genomic estimated breeding value (GEBV) of a single plant. It involves four basic steps: (a) phenotyping and genotyping of the selected individuals from a breeding population, (b) generation of genomic prediction models, (c) model validation on the test population, and d) prediction of GEBV for non-phenotyped individuals and further selection [32]. Unlike MAS, there are no pre-requisites in GS for a prior information about marker linkage, or QTL localizations in the genome and their relative phenotypic effects [33].

## 3. Mutagenesis as a Source of Genetic Variability in Tree Plants

**Figure 3:**
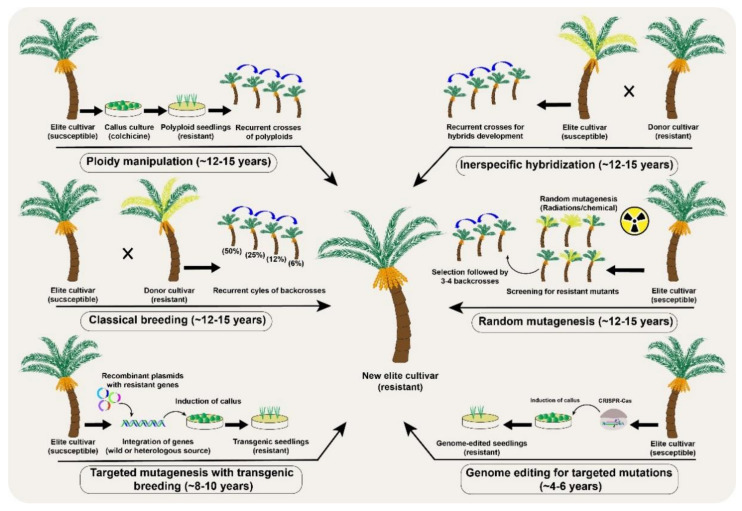
A comparative analysis of different conventional and the new breeding tools (NBTs) to modify desirable genetic modifications in a date palm (*Phoenix dactylifera* L.) fruit crop.

Genetic improvement through conventional breeding necessarily requires recurrent selection cycles in fruit trees [6] (Figure 3). However, a major limitation is the large number of crosses and the development of subsequent filial generations. This is even more challenging in fruit trees such that recurrent selection may take decades of continuous breeding efforts [34]. The lengthy breeding process can be accelerated in fruit frees with more advanced techniques such as MAS and GWAS for QTLs [35]. For example, many quality- and yield-related traits have been improved in apple, banana, mandarin, peach, and strawberry through conventional breeding coupled with mutagenesis, MAS, genetic engineering, MAB and others [6,7,9]. The genetic improvements in fruit trees are, however, progressing at a slower pace, but the availability of pangenomes, broader understanding of genotypic and phenotypic interactions and fast-track breeding may hasten the development of fruit tree cultivars with better genetic makeup.

Genetic improvement through conventional breeding is limited to sexually compatible crop plants [36]. Nevertheless, the genetic diversity of self-incompatible plants can be increased by mutagen treatment (physical or chemical) to induce new mutations in cultured cells, seeds, seedlings, or sometimes whole plants. Random mutations are preferred in seeds because the small number of cells in the developing embryo makes mutagenesis much easier and produces fewer chimeric plants [37]. During somatic mutations, a fewer number of cells or limited sectors in the apical meristem are affected, creating chimeric mutated plants. Such sectorial mutations involve genetic differences in either one or two layers of cells and is more frequent in vegetatively propagated fruit trees [38]. An effective way to dissociate chimerism in vegetatively propagated plant is through organogenesis or embryogenesis [39,40]. The mutation frequency and population structure of the mutants directly depend upon the type of mutagen and the time of exposure. Irrespective of the used mutagens, the ultimate induced mutations are random and therefore require a large screening population to identify the desired mutants [41]. Mutation breeding is advantageous over conventional breeding because it precludes segregation progenies while improving the genetic make-up during selection cycles.

High energy X-ray radiations were the earliest mutagens used to induce desired traits in fruit trees [37]. Currently, X-ray mutagenesis has been either replaced or supplemented with other more advanced physical mutagens such as fast neutrons, ionizing radiations and gamma rays. Besides bringing about beneficial mutations from single-nucleotide replacements to chromosomal aberrations, these mutagens may trigger DNA damage directly or indirectly in the form of oxygen radicals [42]. Physical mutagenesis has been successfully used to improve a number of traits in fruit trees—for example, improved heat tolerance in pineapple [43], self-fertile in cherry fruits, fruit color in apple, bunch size in banana, short-statured papaya plants, disease resistant pear and growth earliness in grapevine [44].

Among the chemical mutagens, ethylmethanesulfonate (EMS) is the most widely employed alkylating agent in fruit crops [45], including banana and peach [46]. However, it is not suitable for vegetatively propagated fruit trees and perennial allogamous fruit trees because of their heterozygous genomes and prolonged life cycle. Although, chemical mutagens are extremely efficient in inducing desirable mutagenesis in whole plants or seeds, it is not recommended for tissue-cultured plants due to their extreme toxicity [37]. Chemical mutagens predominantly cause hemizygous point mutations and successive backcrosses are necessary to obtain a homozygous line and to stabilize the mutated gene of interest [47]. On the other hand, physical mutagenesis has a high risk of a collateral effect on non-targeted genes because the impact of physical mutagens produces multisite mutations of various sizes. For example, the use of fast neutron mutagenesis causes large deletions in the genome besides translocations and chromosomal loss [48,49]. Chemical mutagens are more affordable; however, these carry serious health and environmental risks. Moreover, chemically induced mutations are genetically less stable than physical mutations.

Polyploidy is another interesting natural phenomenon in plant evolution, adaptation, and speciation, which can also be induced using colchicine, for genetic improvements. Colchicine application induces autopolyploidy by blocking mitosis without interfering with DNA replication and ultimately doubles the chromosome numbers (Figure 3). The generation of triploid dessert apple and tetraploid grapevine cultivars are successful examples of autopolyploids in fruit crops [50]. Interspecific hybridizations have also been tested in some citrus cultivars, including the formation of natural hybrids [51]. However, as in conventional breeding, if the hybrids are fertile in perennial fruit trees, multiple backcrosses are still needed to remove the undesirable genetic background of the recessive parent. For example, scab resistance in apple took more than 40 years [52], and the enhancement of sugar and antioxidants levels in elderberries took at least 10 years through the interspecific hybridization approach [53].

Somaclonal variation is a natural phenomenon occurring during in vitro tissue culturing and can produce useful genetic variations in plants [54]. It includes DNA-related genetic or epigenetic variations, which induce phenotypic changes distinguishable from the original parent. Major causes include but are not limited to prolonged in vitro culturing, tissue culturing media composition, the presence of phytohormones and certain other mechanical factors during culturing [55]. Somaclones can be detected through morphological assessments of the off-type regenerants, biochemical response of explants, fingerprinting with protein or isozymes-based markers, and cytogenetic assessment [56,57]. In addition, more advanced DNA- or transposon-based molecular markers [58] and the use of next-generation sequencing (NGS) screening have also been successfully applied to detect somaclonal variations in fruit tree breeding.

### TILLING as a Powerful Tool in Mutation Breeding

Numerous significant genes from older mutant cultivars continue to serve as a foundation for modern gene pools in commercial cultivars. Nonetheless, the burden of unwanted genetic mutations and the development of new breeding tools (NBTs) have had an effect on the use of random mutation techniques [59]. Recent advancements in screening methods enable the detection of SNPs and complex traits at the molecular level, which are otherwise difficult to discern with conventional screening methods. The utilization of mutagenesis underwent a huge change with the development of TILLING (targeting induced local lesions in genomes) as a high-throughput mutant screening technique to identify point mutations at a specific locus in the mutated genome [60]. The TILLING technique redirected mutation breeding away from laborious forward genetics approaches to reverse genetics approaches, allowing plant breeders to detect mutations in known genes. Furthermore, TILLING has been accompanied with the more advanced next-generation sequencing (NGS) techniques to provide more practical solutions to bypass extensive mutant screening for the selected genes [61].

The major mutation screening methods in TILLING include celery nuclease (CEL I) [62], high-resolution melting (HRM) [63] and NGS [64]. The mismatch-specific CEL I method is a popular TILLING technique, which is coupled with the LI-COR gel analyzer system. The HRM incorporates the PCR technique in which the monitoring of dsDNA product is monitored with a dsDNA-specific fluorescent dye followed by the formation of a high-resolution melting curve. The more advanced NGS technique has further facilitated the mutant screening in a TILLING population through whole-genome sequencing, de novo assembly and resequencing tools.

The basic procedure of TILLING includes mutation induction through chemical, physical or biological agents to produce an M1 population. These M1 plants are then allowed to self-pollinate and generate M2 plants. Total genomic DNA is isolated and subjected to eightfold DNA pooling followed by PCR amplification of the gene of interest. The recurrent heating and cooling steps form heteroduplexes, which are then incubated with CEL I endonuclease to cleave mismatches in these heteroduplexes. The cleaved DNA products are separated on a denaturing gel electrophoresis and the fluorescence is detected with a LI-COR DNA analyzer. The induced mutations are then verified by sequencing of the polymorphic individuals, respectively [65]. Although the CEL I -based TILLING platform has been widely used, the critical steps such as enzymatic digestions, cloning and gel electrophoresis make it time consuming. Moreover, insufficient genome sequence information of many plant species affects the efficacy of this TILLING platform [66]. Contrarily, the HRM-based TILLING offers more accurate, sensitive, and cost-effective mutant screening through PCR and analysis of the DNA melting curve. Nevertheless, detection of small insertions and deletions is difficult and limited to amplicons with a size <450 bp with HRM [67]. The NGS-based TILLING platform is comparatively a potential screening method with more accurate mutant screening. However, the high cost, the generation of a large sequencing dataset and the requirement of sophisticated bioinformatics tools still pose major challenges to its adoption in studying the genetics and genomics of mutagenic studies [65].

## 4. Genomics and Genetic Engineering Perspectives of Trees

The genetic improvement of the tree plant genome can be accelerated through two distinct approaches: MAB through quantitative trait loci (QTL) mapping, and direct gene transfer through genetic engineering. The whole genomes of many tree plants have been completely sequenced; consequently, comprehensive genetic architecture of useful genetic traits are now available, which can be helpful for marker-assisted breeding, MAB [68,69]. The availability of such datasets can widely assist in genetic expression, and functional and comparative genomics. Moreover, recent developments in –omics and NGS technologies and, in parallel, more advanced bioinformatics tools, can expedite in-depth molecular studies in trees [70,71]. The transcriptomic, proteomic and metabolomics data sets of woody plants are dynamically bridging the gaps between tree genomes and genetic expression studies.

Genetic transformation can be improvised by inserting single or multiple genes directly into the elite background across the species or genus without long cycles of selections and screening [72,73], e.g., herbicide tolerance in populous [74]. The first application of genetic engineering in fruit trees was in papaya when papaya varieties ‘Sunset’ and ‘Kapoho’ were genetically modified by inserting the capsid protein (CP) gene of papaya ringspot virus (PRSV) to confer viral resistance. Consequently, the first transgenic papaya cultivar was developed in 1998 [75]. Recently, the USA approved a non-browning arctic apple cultivar [76,77]. Several other genetic traits for fruit quality, tree physiology and abiotic stress tolerance have been successfully engineered for transgenic apple, banana, papaya, and pineapple [73,75]. Current transgenic fruit trees approved in the USA include papaya against PRSV, plum against plum pox virus (PPV) [78], apple with the non-browning trait [79] and pineapple cultivar ‘Pinkglow’ [80]. Transgenic papaya plants have been successfully engineered to alter elite traits related to tree growth, nitrogen metabolism, lignin contents and abiotic stress tolerance [81,82]. Moreover, resistance in papaya was also conferred against phytophthora blight, papaya dieback disease (PDBD) and papaya ringspot virus (PRSV) in several studies [83]. Among non-transgenic approaches, dsRNA-mediated protection strategies have also been practiced in papaya against PRSV [84]. Similarly, eucalyptus species have also been genetically transformed to introduce genes from endogenous or heterologous sources to modify their salt tolerance status and secondary cell wall constituents [85]. Many pine softwood tree species have also been utilized for transgenic developments for various traits [69].

## 5. Genome Editing in Precision Breeding

Precision breeding techniques encompass a broad range of technologies that enable the introduction of genetic variation into a plant genome. It combines and utilizes a variety of innovative technologies to engineer desired traits in plants in order to drive new agricultural advancements. In the recent years, the execution of contemporary genome-editing technologies has enabled researchers to easily, swiftly, and economically introduce site-specific modification at the desired DNA sequences in a wide range of cell types and organisms. The CRISPR-Cas based genome-editing technologies can be customized easily to target a desired locus and has brought an unparalleled revolution in agricultural sciences and precision plant breeding [86,87]. CRISPR-Cas tools can foster crop resilience and reduce chemical crop protection with a strong environmental and public health impact on crop production.

### 5.1. Principle and Types of CRISPR-Cas Systems

CRISPR-Cas systems are prokaryotic immune systems that protect cells by selectively and specifically cleaving the nucleic acids of the invaders, such as viruses and plasmids [88]. Since the discovery of the CRISPR system, several new versions of CRISPR-Cas have appeared [89]. Spurred by the interference ability of CRISPR-Cas systems, they are classified into two major classes—I and II—based on the structural variation and arrangement of Cas genes. Both classes are further sub-divided into six main types related to the type of nucleic acid they target [90]. Another benchmark difference between these two classes is the number of nucleases; class I has a single and class II has multiple effector nucleases. Makarova et al. [89] appraised the evolutionary classification of the CRISPR-Cas systems, especially concerning class II and its variants. Among them, the most utilized Cas9 endonucleases are isolated from various microbes such as *Francisella novicida*, *Staphylococcus aureus* and *Streptococcus pyogenes* and belong to class II type II systems [89,90].

**Figure 4:**
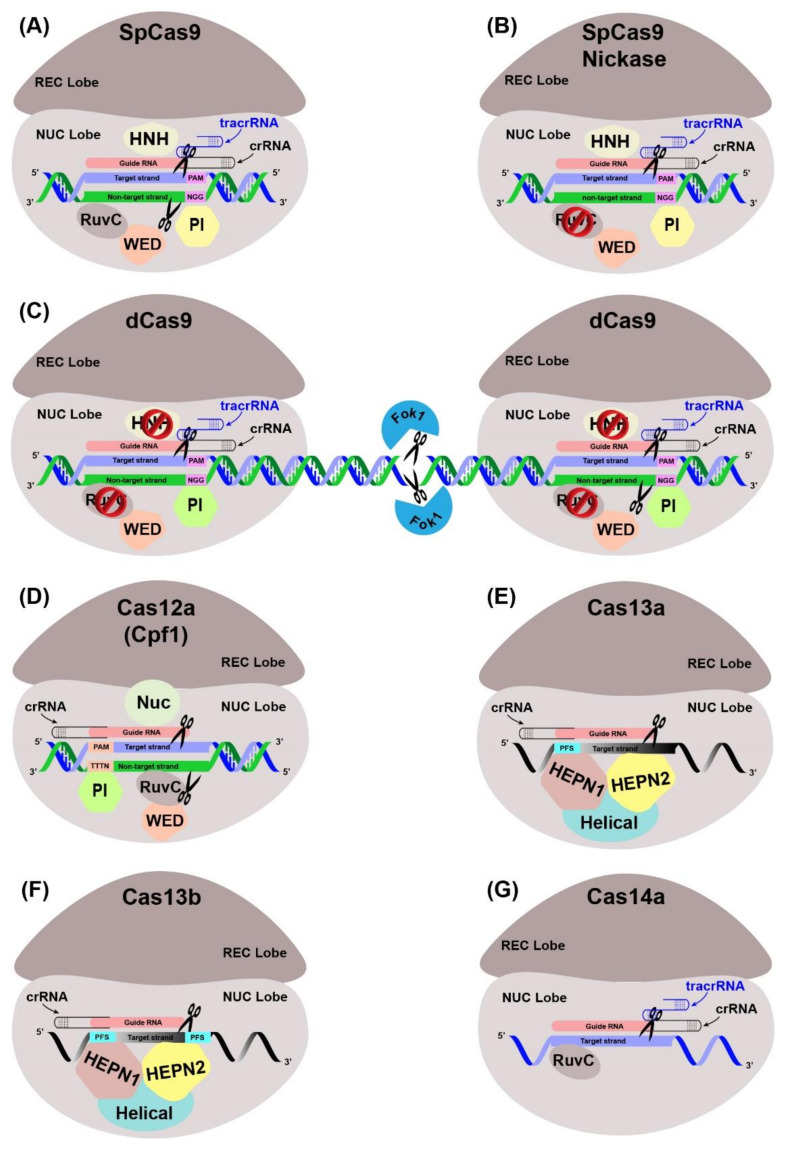
Different Cas protein variants potentially useful for genome editing in perennial trees. (**A**) The CRISPR-Cas9-based system, generally comprised of Cas9, the crRNA, and tracrRNA. It entails hybridization and binding of this triad to the complementary sequence after recognizing a PAM sequence. The recognition lobe (REC) recognizes the crRNA:tracrRNA:target DNA complex, whereas the PAM sequence is intrinsically recognized by the PAM interaction domain (PI) of Cas protein. The HNH and RuvC domains of NUC lobe cleave target and non-target strands upstream of the PAM, respectively, by producing DSB. (**B**) The SpCas9 is engineered to reduce off-target activities by introducing a point mutation in RuvC domain, which results in only a single-stranded break (SSB). (**C**) RNA-guided Fok1 nuclease was fused with an inactive dead Cas9 (dCas9) to enhance on-target efficiency. (**D**) CRISPR-Cas 12a system uses a single crRNA. The Cas12a:crRNA duplex binds to the complementary target sequence with the help of 23–25 nt long gRNA. The Nuc domain cleaves the target strand at ~18 nt downstream to the PAM and the RuvC domain cleaves the non-target strand at ~23 nt downstream of the PAM. (**E**) CRISPR-Cas13a is engineered to target ssRNAs. Instead of a PAM sequence, the binding of Cas13a:crRNA duplex to the target site is mediated via a protospacer flanking sequence (PFS). The REC lobe recognizes the Cas13a:crRNA duplex and the ssRNA substrate is cleaved in a sequence-specific manner through HEPN1 and HEPN2 domains. (**F**) CRISPR-Cas13b has a distinct suppressor and enhancer Cas genes, which are expressed under two PFSs to target ssRNAs. (**G**) contemporary CRISPR-Cas 14a is a single effector system involving a Cas14a protein associated with crRNA and ~130 bp tracrRNA. The hybridization of crRNA and tracrRNA complex is accomplished independent to PAM and RuvC domain cleave the target ssDNA.

The Cas endonuclease function by accompanied CRISPR-RNAs (crRNAs) and trans-activating crRNAs which can recognize foreign nucleic acid sequences [89,91]. The working principle of CRISPR-Cas systems, as a prokaryotic immunity, is based on the integration of the invader’s nucleic acid fragments into the CRISPR locus during infection. Once integrated, the subsequent infections activate the transcription of the integrated fragments and are then recruited by the Cas to cleave the invader’s genome. Different types of Cas nucleases along with their variants, and their specificities, characteristics, and PAM recognitions sites have been summarized (Figure 4, Table 2). The applications of Cas9 have been increased immensely after the invention and customization of single-guide RNA (sgRNA) [92]. Cas9 proteins contain two unique nuclease domains: RuvC and HNH. The former domain cleaves the non-target DNA strand, while the latter domain cleaves the target DNA strand complementary to the sgRNA. The sgRNA is usually comprised of a unique 20 bp sequence and contains a short (usually 2–6 bp in length) essential DNA sequence at the 5′ end and designated as the “protospacer adjacent motif” (PAM) [93,94]. A sgRNA recognizes and binds to the target sequence, and then Cas nuclease induces double-stranded break (DSB) at the target DNA. After the induction of DSB, cell genome repair mechanisms become activated and repair the induced DSB either via non-homologous end joining (NHEJ) or high-fidelity homology-directed repair (HDR) [95]. During NHEJ repair, DSB is directly re-ligated without any homologous template, resulting in insertions, deletions (InDels) or substitutions. On the other hand, HDR is a precise repair pathway that can utilize either an endogenous or an exogenous DNA segment as a template to repair the DSB. HDR may introduce novel alleles, correct existing mutations, or insert a new sequence of interest [96,97].

The class II single Cas protein systems are comprised of Cas9 (type II), Cas12 (type V), Cas13a–d (type VI) and Cas14a–c (type V-F) [98,99]. Among these Cas proteins, Cas9 and Cas12 nucleases target and cleave dsDNA, while different Cas13 (type VI) proteins such as Cas13a (C2c2), Cas13b (C2c6), Cas13c (C2c7) and Cas13d target and cleave the target RNA.

Type I CRISPR systems generally consist of different nucleases, such as Cas5, Cas6, Cas7, Cas8 and Cas11, in a different combination and target dsDNA. Type II consists of Cas5, Cas6, Cas7 and Csf1. Type III is the most abundant group of nucleases present in nearly a quarter of bacterial species and include Cas5, Cas6, Cas8 and Cas10 nucleases [100]. This type is further categorized into two subtypes based on Cas effectors; type III-A and type III-B and include Cas10-Csm and Cas10-Cmr. Another unique feature of type-III systems is their ability to target the nascent mRNAs and DNA sequence in transcriptionally active complexes without the requirement of PAM sequence.

### 5.2. CRISPR/Cas Based GE Strategy in Trees

The use of CRISPR-Cas systems in trees holds unmatched potential for resilience to biotic and abiotic stresses. Until now, these systems have been executed in different trees such as apple, banana, citrus, pear, and others (Table 3). Most of the CRISPR work in trees has been focused on the editing of the easily scorable *Phytoene Desaturase* (*PDS*) gene, which induces albino phenotypes due to reduced photosynthesis and carotenoid synthesis.

### 5.3. CRISPR-Mediated Genome Editing in Apples

Apple (*Malus* × *domestica*) ranks as one of the most produced temperate fruits in the world, with approximately 87 million tons of worldwide production in 2020 [101]. The first successful execution of the CRISPR-Cas system was achieved in the rootstock of the first generation of transgenic apple plants (cv. JM2) by editing the *PDS* gene with up to 31.8% editing efficiency [102]. The CRISPR-mediated GE of *DIPM-1*, *DIPM-2*, and *DIPM-4* genes was achieved in the apple protoplast; however, the indel mutagenesis efficiencies were quite low and in the range of 0.5 to 6.9%, via DSBs [103]. Just a few years later, highly efficient, accurate and DNA-free GE procedures were evaluated in the apple protoplast to modify the PDS gene, based on CRISPR delivery either directly as CRISPR–Cas9 RNP complex or via plasmid. Nonetheless, direct delivery of CRISPR–Cas9 RNPs has a superiority over the plasmid-mediated delivery; direct delivery took about two to three weeks, whereas the plasmid-mediated delivery procedure required more than three months [104]. Initially, Chevreau et al. [105] appraised an adventitious and highly efficient *Agrobacterium*-mediated CRISPR-Cas transformation method in ‘Gala’ apples and demonstrated that the presence of a surfactant (Silwet L-77, 0.002 per cent v/v) in bacterial suspension increased the average transformation efficiency (mean 5.8% and maximum 30%) [105]. Subsequently, the same group targeted two easily scorable genes—*PDS* and *Terminal Flower 1* (*TFL1*)—via CRISPR-Cas9 and a characteristic albino phenotype was observed in 85% and early flowering in 93% of the apple transgenic lines. Sequencing of target zones revealed a variable frequency of mutation, insertions were more frequent than substitutions or deletions, and biallelic chimeras were more prevalent [106]. To deal with the high frequency of chimeras, an adventitious regeneration step from the leaves of T0 transgenic apples was opted. The results yielded 99% truncated alleles of RT0 plants with ~67% of plants having less heterogeneous editing profiles than the T0 [39]. In the same study, a CRISPR-Cas9-based cytidine base editor was successfully executed to modify *PDS* and *acetolactate synthase* (*ALS*) genes in apples.

*Erwinia amylovora*, a causative agent of fire blight disease, is a leading constraint to apple production [107]. It triggers its infection via a DspA/E effector that binds to the apple susceptibility factor, *MdDIPM4*. The knockdown of *MdDIPM4* via a heat-inducible CRISPR/Cas9-FLP/FRT system yielded an editing efficiency of 75% in 57 transgenic lines. Seven GE lines challenged with the *E. amylovora* exhibited a substantial reduction in susceptibility, and almost all of the challenged lines revealed successful elimination of the transgene upon exposure to heat. Another major threat to apple is *Botryosphaeria dothidea*, which triggers the expression of *MdCNGC2* and defense-related genes including *MdPR1*, *MdPR2*, *MdPR4*, *MdPR5*, *MdPR8*, and *MdPR10a* in apples. The CRISPR-Cas9-mediated knockdown of *MdCNGC2* led to a reduction in lesions caused by *B. dothidea* [108].

Apart from induced mutagenesis, a reliable and efficient method for the identification of prevalent viruses/viroids was developed based on a CRISPR-Cas12 platform that could detect most of the viruses in one hour and is highly reliable [109].

### 5.4. CRISPR-Mediated Genome Editing in Banana

Bananas are the fourth largest food crop around the globe, one of the most important commercial fruits in the tropics and cultivated in 130 countries [110,111]. Traditional breeding is challenging in banana due to its complex triploid genome and parthenocarpic nature [112], but modern techniques such as CRISPR-Cas have opened new horizons to tackle such problems. Recently, the CRISPR-Cas system was used to target the conserved domain of two RAS-PDS genes by a common sgRNA in the embryonic cell suspension culture of banana cv. Rasthali. The regenerated plantlets exhibited a range of albino to variegated phenotypes at a 59% mutation rate [113]. The multiplexed CRISPR-Cas approach using polycistronic tRNA-gRNA system was employed in banana cv. Cavendish to target exon1 of the *PDS* gene, which yielded albino phenotypes associated with triallelic deletions or insertions of 19 regenerated plants at a 100% mutation efficiency [114]. The results of these studies demonstrated the feasibility of GE in banana via CRISPR-based targeted genome mutation.

**Table 2:** The characteristics, specificities, and nucleic acid targets of different Cas proteins and their variants.

Cas Type	Organism	Size (Amino Acids)	Class/ Type	PAM Site	Altered PAM	Types of End	Mutations	Plants	References
SpCas9	*Streptococcus pyogenes*	1368–1424	2/II	NGG	--	Blunt/ds or ss	--	Many plant species	[92,115,116,117]
SpCas9 VQR	*S. pyogenes*	1372	2/II	NGA	Yes	Blunt/ds or ss	D1135V/R1335Q/T1337R	Rice	[118]
SpCas9 EQR	*S. pyogenes*	1372	2/II	NGAG	Yes	Blunt/ds or ss	D1135E/R1335Q/T1337R	-	[118]
SpCas9 VRER	*S. pyogenes*	1372	2/II	NGCG	Yes	Blunt/ds or ss	D1135V/G1218R/R1335E/T1337R	Rice	[118]
SpCas9 D1135E	*S. pyogenes*	1372	2/II	NAG/NGA	Yes	Blunt/ds or ss	D1135E	-	[117]
SpCas9 QQR1	*S. pyogenes*	1372	2/II	NAAG	Yes	Blunt/ds or ss	G1218R/N1286Q/I1331F/D1332K/ R1333Q/R1335Q/T1337R	-	[119]
SpCas9-NG	*S. pyogenes*	1372	2/II	NG	Yes	Blunt/ds or ss	R1335V/L1111R/D1135V/G1218R/ E1219F/A1322R/T1337R	*Arabidopsis* and rice	[120]
SpCas9-HF1	*S. pyogenes*	1368	2-II	NGG	Enhanced specificity	Blunt/ds or ss	N497A/R661A/Q695A/Q926A	*Arabidopsis* and rice	[121]
eHF1-Cas9	*S. pyogenes*	1368	2-II	NGG	Enhanced specificity	Blunt/ds or ss	N497A/R661A/Q695A/K848A/ Q926A/K1003A/R1060A	Rice	[122]
HiFi Cas9	*S. pyogenes*	1368	2-II	NGG	Enhanced specificity	Blunt/ds or ss	R691A	Rice	[123]
XCas9	*S. pyogenes*	1368	2-II	NG, GAA & GAT	Enhanced specificity	Blunt/ds or ss	A262T/R324L/S409I/E480K/ E543D/M694I/E1219V	Rice	[124]
dCas9	*S. pyogenes*	1368	2-II	NGG	No	Blunt/ds or ss	D10A/H840A	*Arabidopsis* and *rice*	[125,126]
nCas9	*S. pyogenes*	1368	2-II	NG, GAA & GAT	Enhanced specificity	Blunt/ds or ss	D10A	Rice, tobacco	[127,128]
SaCas9	*Staphylococcus aureus*	1053	2-II	NNGRRT	-	Blunt/ds or ss	-	Tobacco, rice, *Arabidopsis* and citrus	[129]
SaCas9-KKH	*S. aureus*	1053	2-II	NNNRRT	Enhanced specificity	Blunt/ds or ss	E782K/N968K/R1015H	Tobacco, rice, *Arabidopsis* and citrus	[130]
BlatCas9	*Brevibacillus laterosporus*	1092	2-II	NNNNCND		Staggered/ds	-	Maize	[94]
FnCas9	*Francisella novicida*	1629	2B-II	NGG	-	Staggered/ds	-	*Arabidopsis*	[131]
Cpf1 (Cas12a)	*Prevotella & Franscisella*	1300	2-V	TTTN	-	Staggered/ds	-	Many plant species	[132,133]
AsCas12a RR	*Acidaminococcus*	1307	2-V	TYCV & CCCC	Yes	Staggered/ds	S542R/K607R	-	[134,135]
AsCas12a RVR	*Acidaminococcus*	1307	2-V	TATV	Yes	Staggered/ds	S542R/K548V/N552R	-	[135]
LbCas12a	*Lachnospiraceae bacterium*	1228	2-V	TTTV	-	Staggered/ds	-	Many plant species	[134]
LbCas12a RR	*Lachnospiraceae bacterium*	1228	2-V	TYCV & CCCC	Yes	Staggered/ds	G532R/K595R	Rice	[135]
LbCas12a RVR	*Lachnospiraceae bacterium*	1228	2-V	TATV	Yes	Staggered/ds	G532R/K538V/Y542R	Rice	[135]
FnCas12a	*F. novicida*	1300	2-V	TTV, TTTV & KYTV	-	Staggered/ds	-	Rice	[134]
FnCas12a RR	*F. novicida*	1300	2-V	TYCV & TCTV	Yes	Staggered/ds	N607R/K671R	Rice	[136]
FnCas12a RVR	*F. novicida*	1300	2-V	TWTV	Yes	Staggered/ds	N607R/K613V/N617R	Rice	[136]
Cas12b	*Alicyclobacillus*, *Acidoterrestris*, *Bacillus* *Thermoamylovorans*, *A. acidiphilus*	1100–1300	2-VB	TTTN	-	Staggered/ds	-	Many plant species	[137,138]
Cas12X	*Deltaproteobacteria*	<1000	2-V	TTCN	-	Staggered/ds	-	-	[139,140]

The presence of the integrated endogenic banana streak virus (eBSV) in the B plantain genome (AAB) is a limiting factor as it becomes activated under water stress conditions, rendering the plants unsuitable for breeding and dissemination. The eBSV was targeted via CRISPR/Cas9 at a 27% editing efficiency in regenerated plantlets, which remained asymptomatic after exposure to water stress, confirming eBSV obstruction at either transcriptional and/or translational levels [141]. To yield transgene-free GE bananas, PEG-mediated delivery of two different CRISPR-Cas9 systems—a CRISPR-Cas12a plasmid and a CRISPR-Cas9-RNP system—was evaluated in banana protoplast to target *PDS* genes. The results of deep amplicon sequencing revealed that the CRISPR/Cas9 system has higher editing efficiency compared to the other two systems used [142]. The *MaACO1* gene regulates ethylene synthesis and fruit ripening in bananas. The disruption of *MaACO1* via CRISPR-Cas9 in *Musa acuminata* (AAA group, cv. Brazilian) banana delayed fruit ripening both in the field and post-harvest storage conditions up to 60 days [143].

The β-carotene-enriched banana cv. Cavendish was developed using a CRISPR/Cas9 after targeting the fifth exon of the *lycopene epsilon-cyclase* (*LCYε*) gene. Sequence analysis of the edited plants revealed multiple indels in the *LCYε* gene, up to sixfold enhanced accumulation of β-carotene, a severe reduction in α-carotene and lutein levels, without any substantial perturbed agro-morphological traits [144]. To examine the functions of five *MaGA20ox2* genes, implicated in having a role in gibberellic acid biosynthesis and plant height, in banana cv. Gros Michel were edited via CRISPR-Cas9 using embryonic cell suspension cultures. The resultant 152 independent modified transgenic lines contained indels as a major mutation type, low transcription levels of these five genes and the modified banana plants exhibited a significant reduction in height compared to wild-types [145].

### 5.5. CRISPR-Mediated Genome Editing in Citrus

Citrus is one of the top three fruit crops across the globe and a source of many nutrients, principally vitamin C. However, it is susceptible to a plethora of stresses, both biotic and abiotic. Due to its long juvenility, polyploidy, long crossing life cycle and high heterozygosity, conventional breeding techniques have often proved time-consuming and tedious.

The first reported execution of the CRISPR-Cas9 system to target the *PDS* gene in the leaves of sweet orange yielded a mutation efficient of 3.2 to 3.9% with no quantifiable off-targets [146,147]. Subsequently, the same researchers successfully employed CRISPR-LbCas12a to edit the *PDS* in citrus with improved editing efficiency [148]. Modification of the *CsLOB1* canker susceptibility gene in Duncan grapefruit via CRISPR-Cas9 was achieved at a mutation frequency of 23.80 to 89.36% with no off-targets. The regenerated six edited plant lines were inoculated with the pathogen *Xanthomonas citri* ssp. citri, then two of the four edited lines exhibited comparable canker symptoms to wild grapefruit, while the remaining four remained asymptomatic at the beginning, but showed very mild symptoms at the later stage [149].

### 5.6. CRISPR-Mediated Genome Editing in Papaya

Papaya is a tropical fruit of commercial importance due to its high nutritional and medicinal value. In 2019, papaya was grown on 462,552 ha, with a current total world production of 13,735,086 tons [101].

The papaya produces a unique cysteine protease (papain), via regulating *PpalEPIC8* gene to counter the invading oomycete, *Phytophthora palmivora*. Homozygous *PpalEPIC8* mutants were produced via the CRISPR/Cas9, which suggested that *PpalEPIC8* does indeed play a role in *P. palmivora* virulence by inhibiting papain [150]. Another similar study on *P. palmivora* characterized a glycoprotein, *Ppal15kDa*, of *P. palmivora* that is highly induced during infection in papaya plants. Six *Ppal15kDa* mutants produced through CRISPR/Cas9 having homozygous mutation completely lost the pathogenicity, while the heterozygous mutants exhibited varying levels of infection, suggesting that *Ppal15kDa* plays an important role in the normal development of *P. palmivora* infection. Overall, a unique component with a role in the pathogenicity and development of *P. palmivora* infection or possibly other *Phytophthora* spp. was demonstrated in this study [151].

### 5.7. CRISPR-Mediated Genome Editing in Pear

The area under pear cultivation is 1,379,387 ha, with annual production of 13,919,075 tons, which includes both Asian pears (*Pyrus* sp.) and European pears (*P. communis* L.) [101].

Charrier et al. (2019) knocked out *TFL1* genes in pear, which led to early flowering in 9% of the transgenic lines. Sequencing of the target region of transgenic lines revealed that mutations were induced at varying frequencies and insertion mutation was dominant over deletions and substitutions. Nonetheless, the most frequently demonstrated edition pattern of *TFL1* genes was biallelic chimeric. The high frequency of chimerism is a problem, and this was solved by including an adventitious regeneration step from leaves of T0 transgenic pears. In addition, CRISPR-Cas9 BE was executed to induce C-to-T base substitution in pears by co-editing *ALS* and *PDS* genes, which yielded albino and chlorsulfuron lines in pear [39].

In the dwarf pear (*Pyrus bretschneideri*), the tree achieved enhanced yield; the loss-of-function mutant of the *PbPAT14* gene was generated by the CRISPR/Cas9 system. Sequence analysis revealed that out of 22 dwarf yellow lines, six were homozygous mutants and had an elevated level of endogenous abscisic acid (ABA) [152].

### 5.8. Mitigation of Off-Target Mutations in GE Trees

The mitigation of off-target mutations is critical in tree species breeding to bring genetic improvement without disrupting the genetic background of the parent tree. A systematic approach to the design, execution and delivery of the best results is required for successful implementation of the CRISPR-Cas system, without overloading off-targets. In the subsequent sections, key points are addressed.

#### 5.8.1. GC Content of sgRNA

The gRNA structure and its GC contents play a key role in determining the specificity of the CRISPR-Cas system. Ideal GC contents of 40 to 60% in gRNA sequence form a stable DNA:RNA duplex, destabilize off-target binding and ultimately enhance on-target activity [153]. In addition, purine residues at the end of four nucleotides in gRNAs—in particular, guanine at the 20th position and cytosine at the 16th position—improve editing efficiency [154,155]. A positive correlation has been identified between PAM-proximate GC % and gRNA secondary structure.

#### 5.8.2. gRNA Length and Mismatches

The length of the gRNA determines its functionality and the level of off-target activity. Different lengths, 16 to 20 nucleotides long, of gRNAs were evaluated for GE efficiency and off-targets, of which 17-nucleotides-long gRNAs yielded higher GE efficiency compared to 18- to 20-bp-long gRNAs, but 20-bp-long gRNAs did not exhibit any unwanted mutations [156,157]. Dead RNA off-target suppression (dOTS), the new strategy employing dead truncated gRNA, has resulted in reduced off-target activity and increased on-target activity by 40 times [158]. General guidelines for mitigating off-targets have been formulated as: (a) more than three mismatches within the first 7 to 10 bp of the PAM; and (b) gRNA bulges within the first 12 bp of the PAM, which should be avoided [159]. Lee et al. [160] looked at off-target mutations in rice using four sgRNAs and found that the highest off-target mutation rate (67.5%) occurred in the presence of two mismatched bases between the target site and sgRNA; it became severely compromised (2.5%), with six mismatches. Using more than one mismatched RNA base pair to target a sequence can prevent unintended changes in rice plants.

#### 5.8.3. Chemical Modification of gRNA

Chemical modification of gRNA has the potential to improve GE efficiency. A 40- to 120-fold reduction in off-target activity was observed after the incorporation of 20-O-methyl-30-phosphonoacetate into the gRNA ribose-phosphate backbone [161]. The modification of hairpin structure at 50 bp upstream of gRNA enhances the specificity of Cas proteins by reducing off-target effects by up to 55-fold [162].

#### 5.8.4. Concentration of Cas Protein/gRNA

Controlled and low expressions of Cas protein/gRNA could effectively reduce off-target levels. Compared to the constitutive (CaMV35S) promoter, Cas9 expression under an inducible (egg-cell) promoter yielded a high on-target efficiency in *Arabidopsis* plants [133]. Similarly, Cas9 expression under embryo-specific promoters (YAO) yielded improved GE efficiency in *Citrus sinesis* at the reproductive stage [159]. The expression of the Cas9 protein in monocots under the control of plant endogenous promoters resulted in higher on-target mutations than the constitutive CaMV35S promoter [163,164,165,166]. Likewise, comparative studies were conducted to assess the Cas9 expression under endogenous and constitutive promoters; the results showed that the endogenous promoter yielded improved heritability and on-target efficacy [103,167,168,169]. Soya bean promoter (U6-10) and *Arabidopsis* Ubi (AtUbi) promoter-mediated expression of Cas9 protein was investigated in *Glycine max*; a two- to fourfold improvement in on-target efficacy was achieved by the U6-10 promoter compared to the AtUbi promoter [170].

#### 5.8.5. Cas Protein Variants

Aside from the known Cas protein variants, several new versions have been developed through protein engineering to improve the on-target efficiency. Two of the most used Cas proteins—Cas9 and Cas12a—have been shown to be highly efficient, with Cas9 efficiency exceeding 90% and Cas12a efficiency at about 60% [171]. Two naturally occurring Cas9 variants—SaCas9 (*S. aureus*) and StCas9 (*Streptococcus thermophilus*)—recognize longer PAM sequences, including NNGRRT and NNAGAAW, respectively, that can ultimately enhance their on-target efficiency. SpCas9, however, demonstrated higher on-target specificity and expression levels compared to SaCas9 in *A. thaliana* [172]. Two engineered versions of the Cas9—SpCas9-VQR and SpCas9-EQR (Table 3)—were tested in plants for their on-target efficiency and found to be more efficient than the conventional Cas9 [173].

Another simple yet robust approach to mitigate the off-targets is to use a Cas9 mutant paired with a nickase (HNH or RuvC-like). The ability of Cas9-paired nickase to reduce unwanted mutations is its main advantage over Cas9. Other variants of Cas proteins referred to as ‘deactivated/dead (d) Cas’ have been developed by mutating the nuclease domain, and these ‘dead’ variants have been widely used in GE [174]. The dCas protein variants bind to the target sequence to block transcription elongation [175]. The recently developed base editors (ABE and CBE) can convert G to A and C to T in the target genome, while the CRISPR system equipped with deaminases can regulate gene expression [176].

**Table 3:** Execution of CRISPR-Cas systems in different fruit trees (correct to alpha order of tree species).

Tree Species	Gene Target	Trait Modified	CRISPR-Cas System	Reference
Apple	*PDS*	Albino phenotype	CRISPR-Cas9	[102,104,106]
	DIPM-1 DIPM-2 DIPM-4	Fire blight	CRISPR-Cas9	[103,107]
	*TFL1*	Early flowering	CRISPR-Cas9	[106]
	*ALS*		CRISPR-Cas9	[39]
	*CNGC2*	*B. dothidea* resistance	CRISPR-Cas9	[108]
		Detection of viruses and viroids	CRISPR-Cas12	[109]
Banana	*PDS*	Albino phenotype	CRISPR-Cas9	[113,114]
	*PDS*	eBSV resistance	CRISPR-Cas9	[141]
	*PDS*	Albino phenotype	CRISPR-Cas9	[142]
	*PDS*	Albino phenotype	CRISPR-Cas12a	[142]
	*ACO1*	Fruit ripening	CRISPR-Cas9	[143]
	*LCYε*	β-carotene	CRISPR-Cas9	[93]
	*GA20ox2*	Gibberlic acid biosynthesis	CRISPR-Cas9	[145]
Cacao	*TcNPR3*	Resistance to *Phytophthora tropicalis*	CRISPR-Cas9	[177]
Citrus	*PDS*	Albino phenotype	CRISPR-Cas9	[146,147]
	*LOB1*	Canker resistance	CRISPR-Cas9	[149]
	*PDS*	Albino phenotype	CRISPR-Cas12	[148]
Papaya	Papain (*PpalEPIC8*)	Cysteine protease, *P. palmivora* resistance	CRISPR-Cas9	[150]
	*Ppal15kDa*	*P. palmivora* resistance	CRISPR-Cas9	[151]
Pear	*TFL1*	Early flowering	CRISPR-Cas9	[106]
	*PDS* and *ALS*	Albino and chlorsulfuron	CRISPR-Cas9 C-to-T BE	[39]
	*PbPAT14*	Dwarf and yellowing	CRISPR-Cas9	[152]

### 5.9. CRISPR Delivery Techniques and Vectors

Delivery of the CRISPR-Cas system is one of the crucial elements for its successful execution. Several transformation techniques to deliver the CRISPR system into plants are practiced: *Agrobacterium*-mediated transformation, biolistic transformation, RNP-complex, polyethylene glycol (PEG)-mediated transformation, lipid and polymer transformation, and viral vectors.

PEG-mediated transformation of the CRISPR-Cas system was initially achieved in maize [166]. Since then, several plant species have been successfully transformed [178]. The main challenges of using PEG-mediated delivery systems are the isolation of suspension cells and protoplasts.

*Agrobacterium*-mediated transformation is the most common method of delivering the CRISPR-Cas system into plants. This technique has offered improved transformation efficiency rate (40% to 100%) compared to particle bombardment in the plant species [114,179,180]. *Agrobacterium*-mediated transformation via the floral dip method has also been executed in *A. thaliana* [181], *Brassica rapa*, flax, tomato, radish, *Setaria viridis* and wheat [182,183,184,185].

The second most common method of CRISPR-Cas transformation into plants is by biolistic means, which has been executed in a variety of plant species such as brassica [186], maize [187], potato [188], soybean [189] and wheat [153]. However, the regeneration of transformed tissues, optimization of selection pressure, time-consumption, less cost-effective and low transformation efficiency are the challenges associated with this technique. For example, in maize, merely 2.4 to 9.7% GE efficiency could be achieved via biolistic inoculation [187].

The recently used RNP, a technique for achieving GE plants, includes apple [103], banana [142], brassica [186], capsicum [190], rice [191], potato [188], and lettuce [192]. In RNP-mediated delivery, Cas9 swiftly becomes degraded after cleaving the target site, thus reducing the off-targets vulnerability and GMO/ethical concerns. Six *polyphenol oxidase* genes of mushrooms were edited successfully via RNP-mediated CRISPR delivery, and transgene-free GE mushroom had a 30% reduction in the enzyme activity responsible for browning and also escapes US regulations [193]. This technique is useful for vegetatively propagated trees, where removing transgenes from GE plants via backcrosses is almost impossible.

Viral vectors, both DNA and RNA, have successfully conveyed the CRISPR/Cas9 system to plants. The DNA viruses employed include bean yellow dwarf virus (BeYDV, [194,195], wheat dwarf virus (WDV, [196] and cabbage leaf curl virus (CabLCuV [197], but also RNA viruses including tobacco rattle virus (TRV, [198]. BeYDV vectors yielded a 12-fold improved on-target efficiency in wheat, whereas WDV vectors yielded a 10-fold enhanced on-target efficiency in wheat. The TRV-based vectors lead to 15% fewer off-target mutations.

Several other delivery methods using CRISPR have been reported including cell-penetrating peptides [199], DNA nanoclews [200], Cas9En-arginine nano-assemblies [201], and polyethylene imine (PEI)-based nanocarrier [202]. Future delivery methods based on lipids and polymers will shape CRISPR-Cas technology in the coming years.

### 5.10. Genome Editing Tools: Base, Prime and RNA Editors

The base editing tool is a new innovation in the CRISPR-Cas precise engineering toolbox. Such base editor (BEs) systems use facets of DNA modifying enzymes (such as deaminases) to substitute a nucleotide base. Various versions of CRISPR-Cas systems are available for all four transition mutations, such as C to T, G to A, A to G, and T to C. BEs are categorized mainly as CBEs, which can convert C to T; ABEs, which convert A to G; and RBEs, which convert A to I, or C to U. BEs are comprised of four main components—gRNA, nCas or dCas, a deaminase, and a uracil DNA glycosylase inhibitor (UGI). The function of the gRNA is to guide a CRISPR-Cas9 to bind to the target sequence, after which the Cas9 catalyzes the base conversion [203], while UGI is a bacteriophage-derived 83-residue protein that blocks the uracil DNA glycosylase activity. During substituting a base via the CRISPR-Cas system, DSB is not induced, so the cell’s DNA repair mechanisms are not activated, resulting in considerably fewer off- and on-target indels [204].

#### 5.10.1. Base Editors

Adenine base editors (ABEs) yield much cleaner DNA products than cytosine-BE and they are less prone to insertions and have virtually no inversions. A·T to G·C conversions have been successfully mediated in various plant species [205,206,207,208]. In high-quality sequencing of ABE-edited wheat and rice target DNA, the ABE-P1 system led to no undesired off- and on-target base editing [205,207]. Similarly, four cotton genes for *GhCLA* and *GhPEBP* were targeted using a unique base editor *Gossypium hirsutum* (Gh) BE3, resulting in cleavage efficiencies ranging from 27 to 58% with only 0.1% off-target activity [209].

An adenine base editor (Adenine base Editor14) based on nCas9 (D10A) that is guided by TadA:TadA7.10 heterodimer was engineered to achieve A·T to G·C conversion in *OsMPK6*, *OsSERK2* and *OsWRKY45* in rice with editing efficiencies of 16.7, 32.1, and 62.3%, respectively [210]. A new plant-based ABE (based on an evolved tRNA adenosine deaminase fused to the nCas9) enabled A·T to G·C editing in protoplasts and regenerated rice and wheat at frequencies up to 7.5 and 59.1%, respectively. A rice mutation *ACC-T1* with C2186R mutation conferred herbicide tolerance [205]. Four chimeric ecTadAs were made after fusing *E. coli* TadAs, which had different modification, with D10A. The plant ABE-P1 (plant version) produced by fusing recombinant ecTadA*7.10 protein to the N terminus of nCas9 (D10A) yielded 26 and 12.5% editing efficiency in the *OsSPL14* and *OsSLR1* gene, respectively. In addition, this system has enabled multiplex base editing in *Arabidopsis* and *B. napus* with respective efficiencies of up to 4.1 and 8.8% [208]. New ABEs were developed by engineering the SpCas9 variants to expand the target sites. This led to an increase in *OsSPL14* and *OsSPL17* editing efficiency of 25 and 45%, respectively, of the rice genome [208].

The high frequency of SNPs in tree genomes makes them good candidates to execute BE systems. A majority of plant resistance genes are allelic and may differ by a single or a few nts. Resistance to several pathogens can be engineered by substituting these nts via BEs. Likewise, BEs can be employed to engineer the susceptibility (S) genes to generate resistant alleles. However, such modifications may lead to pleiotrophic effects, such as reductions in yield, growth, or other stresses. To circumvent this issue, nts in the promoter region can be engineered though BEs to enhance resistance without compromising its pleiotropic effects.

#### 5.10.2. Prime Editing

Prime editing (PE) is the latest GE technology that has enabled almost all types of edits, including transitions (C→T, G→A, A→G, T→C) and transversion mutations (C→A, C→G, G→C, G→T, A→C, A→T, T→A, T→G), and small indels, without the requirement for inducing DSBs [211,212]. The prime editing system has two main components: a prime editing guide (peg) RNA and a prime editor. A short 8- to 16-nucleotide primer binding site (PBS) sequence, a corresponding reverse transcriptase (RT) template, and a desired editing sequence serve as the foundation for the construction of a pegRNA. As prime editing offers a great deal of flexibility for achieving a variety of genome edits, it offers tremendous potential for the advancement of superior crops for a wide range of purposes, such as increasing yield, resisting various abiotic and biotic stresses, and improving the quality of plant product.

#### 5.10.3. RNA Editing

RNA editing (RE) is another modified version of BE to regulate RNA splicing pathways. The majority of the eukaryotic mRNA is spliced according to the GU/AG rules, with 5′GU serving as the splice donor site and 3′AG as splice acceptor site. Any mutation at these sites can result in mis-splicing or loss of a certain splice form. By adopting the same strategy, G was substituted for A in the splice donor site via the RE to hamper the excision of an intron to gain hypersensitivity to abscisic acid [213]. In a similar study, single null mutants of *Arabidopsis* MTA genes and double null mutants of rice genes *OsGL1* and *OsNAL1* were generated by mis-splicing [214]. Therefore, CRISPR-Cas BEs could have a substantial role in tree GE.

PE was used to confer herbicide resistance by targeting three loci of the *Acetolactate Synthase* gene (*OsALS*) in rice. The regenerated rice shoots carrying either ALS-PE2 or ALS-PE3 were herbicide resistant, and Sanger sequencing revealed the successful editing [215]. Mutations induced by PE can be variable and one type of mutation can occur at a higher rate than others. Reportedly the frequency of deletions (6 bp) ranges up to 21.8% [216] and insertions (3 bp) range up to 19.8% [217], while the mutation frequency ranges from 0.03 to 18.75% in rice [218]. Mutations in wheat were less common than in rice, particularly at the codon level, which is about 1.4% in comparison with 9.38% in rice. For all cases with 12 base-to-base substitutions, the frequency of edits was between 0.2 and 8% [218]. In plants, the frequency of indels increases as the length of targeted sequences increases.

The BE system could also be used to understand the role of conserved amino acids in protein structure and function. Using the CBE, the role of four *Arabidopsis* genes was revalidated as either constitutive splicing or impeding it.

## 6. Techniques to Estimate and Quantify the Mutation Rate

Despite remarkable progress in GE, current methods to detect mutations induced by CRISPR-Cas systems are still challenging in plants. Detection techniques are extremely important when inbred lines are desired, or when screening a large population. Nevertheless, several methods have been applied to detect both on- and off-target mutations. The methods share certain pros and cons; some of the most commonly used methods are briefly discussed here.

### 6.1. T7E1 Mismatch Cleavage Assay

The T7E1 mismatch cleavage assays are popular due to their speed, cost, simplicity and effectiveness on single clones and pooled samples. This technique is used to identify clones and clone segments prior to more detailed analysis. This assay relies on the hybridization of modified and wild-type DNA strands to detect mutation. Mismatches are detected and cleaved by the nuclease enzyme, and the resulting DNA fragments are resolved by gel electrophoresis. However, the assay shares certain limitations, including the lack of sequence information, missing SNPs and small indels [219], possibly requiring optimization, and needing a PCR step to detect homozygous mutations. This technique has been successfully executed in different plant species to detect CRISPR-mediated mutagenesis [220,221,222,223].

### 6.2. High-Resolution Melting Assay

High-resolution melting (HRM) analysis is based on the post-real time PCR (fluorescent dye-based) method and involves the analysis of melting curves. Fluorescent intensity is plotted against the melting temperature to provide raw melt curve data, and each type of genome change generates a unique melting curve. The resulting melting curve helps to distinguish between different mutants such as heterozygous, bi-allelic, or homozygous mutations. HRM is sensitive enough to detect even a single-base indel pair with high precision [224]. HRM analysis requires a simple set-up and allows rapid, high-throughput mutation screening, but requires a dedicated software. This particular technology has been found to work across different plant species [225,226,227,228,229].

### 6.3. Sanger Sequencing

This method of identifying induced mutations at the target locus involves amplifying the target region by PCR, followed by a Sanger amplicon sequence. This method is simple, robust, cost-effective and provides information about the type and frequency of mutations. However, in order to identify mutations in all copies of the genome, many colonies must be sequenced. Furthermore, Sanger sequencing can be difficult, laborious, and time-consuming. The Sanger sequencing platform is the most widely opted for mechanism of mutation detection after executing the CRISPR-Cas and has been successfully executed in a variety of plants [30,230,231,232,233,234].

### 6.4. Next-Generation Sequencing

Next-generation sequencing (NGS) is a highly robust technique that allows indel detection and simultaneous screening of off-target mutations in both mixed populations and clonal cell lines. These abilities have made it a popular choice among researchers. NGS can detect mutations with a high sensitivity of as low as 0.01% [235] and can detect the locations of indels and whether a cell population is truly monoclonal. The main limitations of NGS are cost, the need for bioinformatics tools, and the production of short readings that can be missed by larger indels. NGS has been successfully utilized in plant species to achieve a comprehensive understanding of all the on- and off-targets present in the edited genome [236,237,238,239,240,241].

### 6.5. FLA-PCR (Fragment Analysis)

Fragment analysis is a capillary electrophoresis (CE) method based on the detection of AFLP, multiplex ligation-dependent probe amplification (MLPA) and SNPs. CE is a proven sensitive, high throughput and high-resolution nucleic acid analysis system. Several fragment analysis methods such as IDAA, fluorescent PCR, and CRISPR-STAT have recently been developed in CRISPR/Cas9 genome editing studies. It has been reported that their sensitivity and resolution are comparable to NGS with an indel detection sensitivity of approximately 0.1% [242]. The bottleneck for fragment analysis methods lies in secondary data analysis, requiring sophisticated software for targeting efficiency calculations for genome editing studies. FLA-PCR has been successfully carried out in some plant species to accurately identify on- and off-targets [243,244].

## 7. Critical Assessment of CRISPR-Cas Based GE in Fruit Trees

Despite the broad applications, and unparalleled popularity and acclaim over other GE techniques of CRISPR-Cas, it still poses some limitations in woody plants. These may include a low mutation efficiency/rate, unwanted (off-target) mutations, inefficient gene delivery techniques, in vitro regeneration dependency, persistent activity in subsequent generations, the spread of transgene to other plants and reversion of mutation via cross-pollination, difficulties of execution in woody plants and long-life cycle, genotypic chimerism and strict GMO regulations. Most of these issues can be tackled through a meticulous approach by avoiding erroneous gRNA design, choosing the best Cas protein variant, designing a better expression cassette (including a promoter), employing a highly efficient DNA delivery approach and targeting the right tissue types [245,246,247].

Tree genomes are highly complex due to their high gene copy numbers and ploidy levels, so knocking out all copies of a gene or gene with high homology is a daunting task. To address such issues using conventional genetic manipulation techniques, a series of allelic mutations is performed first, followed by selection in the segregating population [248]. On the other hand, CRISPR-Cas-mediated mutagenesis has simplified the process of modifying and introducing multiple traits into polyploid plants without introducing any linkage drag. CRISPR/Cas-based knockout mutants have created genome-wide deletion mutants that can be used to study gene function in species that are long lived, cannot be easily self-pollinated, and have low transformation efficiency. The elimination of a binding site for bacterial-coded pathogenesis protein in the citrus genome [147], the prevention of floral development [106,249], and the reduction of lignin biosynthesis [250] have opened up new avenues for the development of genes involved in wood structure and chemistry.

To successfully execute CRISPR-Cas in trees, prior knowledge of chromosomal rearrangements, copy number and genetic variations, indels, SNPs, and transposon occurrence are prerequisites. Creating knock-in mutants of a desired gene by deleting a repressor-binding site in the promoter or by mutating a motif involved in rapid degradation is plausible. Such mutants could be generated to increase resistance to herbicides, insects, or pathogens, and thus provide some of the most promising opportunities to generate value traits for forestry. Furthermore, a single insertion into the target loci (hemizygosity) via DSBs may be advantageous in generating the same gene in the corresponding locus of unrelated genotypes. This approach would allow the production of homozygous offspring in just one generation as two hemizygous insertions at the same locus would be crossed. This would streamline inheritance, mitigate linkage drag, and reduce the inbreeding depression that could otherwise occur from repeated use of the original resistance event.

For most tree species, transformation and regeneration remain major bottlenecks after GE. Lowe et al. [251] demonstrated effective transformation in extremely difficult plant genotypes by overexpressing the morphogenic genes *Baby boom* (*Bbm*) and *Wuschel2* (*Wus2*) from maize. It is anticipated that the same technology could be applied to forest trees after achieving the essential customization. The regeneration bottleneck could be alleviated substantially using transgenes that can boost the regenerability of transgenic cells. The effectiveness of morphogenic genes to induce regeneration in angiosperms was first documented in dicots, where somatic embryos were induced on various explants [252,253], and these genes exhibited improved regeneration [254,255].

Although CRISPR is a precise and powerful GE technique, it creates chimeric plants. Chimerism often occurs in regenerated plants during genetic transformation by organogenesis and it is not eliminated by subsequent selfing in most tree plants due to self-incompatibility, which is controlled by a single S-locus [256]. This phenomenon is further exacerbated by the lengthy and difficult regeneration process during the Cas9 use. One of the important ways to prevent chimerism is to exclude the sexual propagation of transgenic plants [257]. Unfortunately, this step does not apply to a wide range of tree species. Alternative strategies to combat chimerism include adventitious shoot regeneration [258], which has been effectively applied to apples and pears [39,106].

Another major bottleneck to GE plants is troublesome GMO regulations, people, and market trends. Market limitations pertaining to forest industries are now mostly covered by forest certification research. At present, there is limited commercial utility of GE in trees due to marketing and commercialization obstacles [259].

## 8. Biological and Regulatory Constraints to GE in Trees

**Figure 5:**
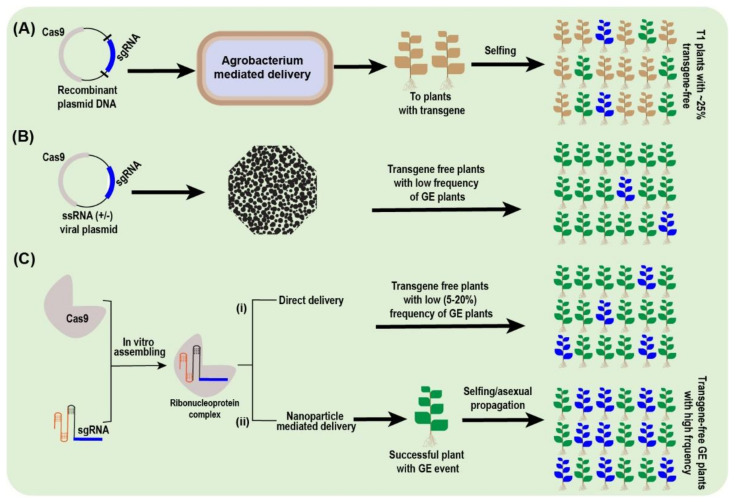
Potential transgene-free approaches to modify tree plants through CRISPR-Cas system. (**A**) Agrobacterium-mediated CRISPR-Cas system keep majority of the transgene cargo in T0 generation. Nonetheless, after selfing, a quarter of the T1 progeny will be transgene free if these plants received a single copy of the transgene. As more than one transgene is inserted by this approach, less than 25% of plants will be transgene free in the T1 generation. (**B**) Viral vector-mediated CRISPR-Cas delivery can lead to yielding transgene-free GE plants. However, their cargo and target specificity limit their usage. (**C**) Ribonucleoprotein (RNP) complex utilizes in vitro hybridization of expressed Cas9 and sgRNA followed by delivery either by biolistic means (i), or via nanoparticles (ii). The resultant cells (both transformed and untransformed) will form transgene-free seedlings, which can be separated out after laborious screening. The wild-type plants are shown as dark green, GE plants harboring transgene as light brown, and GE and transgene-free plants in dark blue color.

Applications of GE in fruit tree breeding have to overcome certain biological and regulatory constraints. The identification of the genetic basis of desirable traits is still a laborious task and involves many forward and reverse genetics tools, along with the execution of whole genome sequence using the NGS approach. After successfully identifying the desired genes, the next challenge is to choose a suitable delivery method of the CRISPR-Cas system and regeneration of GE mutants. The most commonly opted for methods are *Agrobacterium* or viral-mediated systems. Tissue culturing is the most common method for the transformation and regeneration of GE plants; however, many trees lack an established system for efficient transformation and tissue culture. For example, date palm is a major fruit tree in oasis agriculture and due to its large and complex genomic architecture, the application of GE can be a challenging task. Recently, a generalized GE strategy in date palm, its potential applications and limitations have been discussed in detail [260]. Another challenging task is eliminating the footprints of foreign DNA fragments (such as T-DNA insertion from the plasmids) from the GE plants in heterozygous or vegetatively propagated plants [261]. Possibly, the transgene-free mutants can be obtained by high-throughput screening of a large population of the transformants [227] (Figure 5). The transgene-free GE can also be executed by in vitro expression of ribonucleoprotein complexes [262] and transcripts [263]. An alternative to in vitro agro-transformation is *in planta* transformation of plants, which involves targeting in vivo explants (apical meristem, inflorescence, pollen, stigmatic tissue). This method can be further optimized for recalcitrant tree species in classical ways of genetic transformation. In addition, several other key factors can also affect the execution of a GE event in a tree species. These may include the size of sgRNAs and their GC contents, co-expression of sgRNAs and Cas9 protein and the formation of secondary structures during pairing of sgRNAs with the target region [264]. From a scientific perspective, the use of non-inherited and transiently expressed RNP complex to generate DNA-free GE plants is equivalent to using gamma rays or EMS to induce mutations. It is rather advantageous to use GE in contrast to gamma rays or EMS, which are hazardous to human health.

Advancements in GE technologies have enabled researchers to replicate classical breeding outcomes by precisely mutating the genome of many fruit trees (without any off-target loads) and avoiding prolonged cycles of backcrossing and screening. This juridical capability of GE provides an opportunity for GE plants to circumvent the current GMO regulatory framework [47]. In CRISPR-Cas9-based GE in plants, the predominant repair pathway employs NHEJ, which helps to create transgene-free plants because no foreign elements are involved [265]. Moreover, it seems absurd to consider interspecific hybridization more natural than precisely editing the genome for a specific trait using wild plant sources, which leaves no genetic footprints from a foreign source. The regulatory framework model is either product-based (Canada) or process-based (EU); thus, there are different perspectives about the regulatory framework of GE plants (Table 4). For example, Canada imposes a pre-market assessment for any GE plant, feed, or food product, which differs from an already available source [266]. Recently, Canada has announced that it will reconsider its risk assessment policy to exempt GE crops without any foreign DNA footprints from biosafety regulations. Meanwhile, Japan has approved the world’s first GE tomato containing a high level of gamma aminobutyric acid (GABA) (https://www.isaaa.org/kc/cropbiotechupdate/article/; accessed on 7 March 2021). This clearly shows Japan’s policy towards GE crops, which will not be considered as genetically modified. Additionally, Australia permits the use of transgene-free GE crops [267]. In contrast, the European Union Court of Justice has imposed strict GMO regulations on all GE crops [268,269]. Thus, now is an appropriate time to review and revise the global scientific consensus and regulatory framework that are limiting the development of GE cultivars.

## 9. Conclusions and Future Perspectives

Mutation breeding has significantly contributed to crop improvement across the globe and led to the commercialization of hundreds of mutated crops with higher yield potential, improved nutritional quality and tolerance to biotic and abiotic stresses. Mutations have generated impressive genetic resources for all major crops worldwide. Stable gene-specific mutations are now very efficient with the discovery of TILLING as a high-throughput mutant screening technique. Mutations can be more precisely detected at specific loci or genes with TILLING screening based upon CEL I-, HRM- and NGS-based approaches. Under such circumstances, the conventional mutation breeding can be comparable to the NBT based upon a CRISPR-Cas approach. Apart from this convergence, GE may surpass TILLING-based spontaneous and induced mutagenesis approaches due to precision and off-target mediation. Nonetheless, breeding of trees poses a crucial bottleneck such as long-life cycle, the ploidy level, occurrence of sequence polymorphisms, nature of parthenocarpic fruit development, and linkage drag. The development of the NBTs with a high-degree of precision, robust selection, and speed breeding, such as CRISPR-Cas and its contemporary versions (BE and PE), could substantially meet the growing food security challenges. Apart from these, several techniques have been devised to mitigate the off-targets and other limitations of NBTs in trees, such as chimeras. CRISPR-mediated tree breeding has the potential to substantially sustain yield with less effort and cost. Although GE has been accomplished for many field crops, its application in tree breeding necessarily requires the identification of major breeding traits, after communicating with all stakeholders. Moreover, the selection of suitable GE reagents and protocols to regenerate plant mutant requires more suitable methods. Given that, we may speculate that such challenges will be properly addressed in the coming period of time.

## Figures and Tables

**Table 1:** Country-wise varietal approval developed by mutagen treatment.

Common Name	Botanical Name	Variety	Mutagen Source	Country	Year of Registration
Almond	*Prunus dulcis* Mill.	Supernova	Gamma rays (30 Gy)	Italy	1987
Apple	*Malus pumila* Mill.	Mori-hou-fu 3A	Gamma rays (30 Gy)	Japan	1963
		Senbatsu-Fuji-2-Kei	Gamma rays (60 Gy)		1985
		Belrene	EMS	France	1970
		Blackjoin BA 2 520	Gamma rays (50 Gy)		1970
		Courtagold			1972
		Courtavel			1972
		Lysgolden			1972
		Donghenghongpingguo	Gamma rays (250 Gy)	China	1987
		Dovar	X-rays (30–35 Gy)	Netherlands	1978
		Golden Haidegg	Gamma rays (50 Gy)	Austria	1986
		James Grieve Double Red	Gamma rays (62 Gy)	Czech Republic	1995
		McIntosh 8F-2-32	Gamma rays	Canada	1970
		Shamrock	Gamma rays	Canada	1986
Apricot	*Prunus armeniaca* L.	Early Blenheim	thermal neutrons (thN)	Canada	1970
Banana	*Musa paradisiaca* L.	Klue Hom Thong KU1	Gamma rays (25 Gy)	Thailand	1985
		Novaria	Gamma rays (60 Gy)	Malaysia	1995
		AL-BEELY	Gamma rays	Sudan	2007
		Pirama 1	Gamma rays (30 Gy)	Indonesia	2019
		Fuxuan 01	Gamma rays	China	2005
Clementina	*Citrus celementina* L.	Nero	Fast neutron (6 Gy)	Spain	2006
		Neufina			2010
		CLEMENVERD	Fast neutron (5 Gy)	Spain	
Ficus	*Ficus benjamina* L.	Golden King	X-rays (25 Gy) AND Gamma rays (20 Gy)	Belgium	1980
		Golden Princess			
Fig	*Ficus carica* L.	Bol (Abundant)	Gamma rays (50–70 Gy)	Russian Federation	1979
Grapefruit	*Citrus paradisi* Macf.	Rio Red	Thermal neutrons (thN)	United States	1970
		Star Ruby			1984
Indian Jujube	*Ziziphus mauritiana* Lamk.	Dao tien	MNH (0.02–0.04%)	Viet Nam	1986
		Ma hong			
Japanese pear	*Pyrus pyriforia* Nak.	Gold Nijisseiki	Gamma rays (0.12–0.15 Gy)	Japan	1991
		Kotobuki Shinsui	Gamma rays (80 Gy)		1997
		Osa Gold			1997
Lemon	*Citrus limon* L.	Eureka 22 INTA	X-rays (10 Gy)	Argentina	1987
Loquat	*Eriobotrya japonica* L.	Shiro-mogi	Gamma rays (200 Gy)	Japan	1982
Mandarin	*Citrus reticulata* L.	Zhongyu 7	Gamma rays (100 Gy)	China	1985
		Zhongyu 8			1986
		Hongju 420			
		NIAB Kinnow	Gamma rays (20 Gy)	Pakistan	2017
		PAU Kinnow-1	Gamma rays (30 Gy)	India	2017
Mulberry	*Morus alba* L.	Sangfu 1	Gamma rays (75 Gy)	China	1974
		Fuzaofeng	Gamma rays (5 Gy)		1992
		Ji 7681	N2 laser		1988
		Fusang 10	Gamma rays		1980
		Shansang 871	Gamma rays (60 Gy)		1994
		Shigu 11-6	Gamma rays (100 Gy)		1995
		Lala Berry	Colchicine	Japan	2003
		Pop Berry	Colchicine		2004
		S54	EMS	India	1974
Orange	*Citrus sinensis* L.	Hongju 418	Gamma rays (100 Gy)	China	1983
		Xuegan 9-12-1			
		Valencia 2 INTA	X-rays (20 Gy)	Argentina	1987
		IAC 2014	Gamma rays (40 Gy)	Brazil	2016
Papaya	*Carica papaya* L.	Pusa nanha	Gamma rays (150 Gy)	India	1987
Peach	*Prunus persica* L.	Magnif 135	Gamma rays	Argentina	1968
		Shaji 1	CO_2_ laser	China	1985
		Shaji 2			
		Fuku-ekubo	Gamma rays (30 Gy)	Japan	1996
		Shimizu Hakutou RS			2004
		Plovdiv 6	Gamma rays (10 Gy)	Bulgaria	1981
Pear	*Pyrus communis* L.	Fuxiangyanghongdli	Gamma rays (2.5 Gy)	China	1983
		Chaofu 1			1989
		Chaofu 10			
		Chaofu 10			
		Chaofu 2			
Plum	*Prunus domestica* L.	Spurdente-Ferco	Gamma rays	France	1988
Pomegranate	*Punica granatum* L.	Karabakh	Gamma rays (50–70 Gy)	Russian Federation	1979
		Khyrda			
Sour cherry	*Prunus cerasus* L.	Plodorodnaya Michurina	X-rays	Russian Federation	1977
		Karlik Samorodka	Gamma rays		1979
		Polukarlik Orlovskoi Rannei			
		Polukarlik Turgenevki			
		Nishina Zao (DT2008)	Ion beams	Japan	2009
Sweet cherry	*Prunus avium* L.	Compact Lambert	X-rays (40 Gy)	Canada	1964
		Compact Stella 35B-11			1974
		Van 2D-14-11			1972
		Lapins	X-rays		1983
		Lambert 2B-17-18-EC	X-rays (50 Gy)		1972
		Stella			1968
		Stella 16A-7			1972
		Sunburst			1983
		Sumste Samba	Gamma rays		2000
		ALDAMLA	Gamma rays (25 Gy)	Turkey	2014
		BURAK	Gamma rays (50 Gy)		
		Burlat C1	Gamma rays	Italy	1983
		Nero II C1			
		Ferrovia spur	X-rays (4 Gy)		1992
		Super 6	Colchicine	Japan	1997
		Roman Nishiki			2002

**Table 4:** Genome editing related regulations in the selected countries.

Country	Cartagena Protocol of Biosafety (CPB) Status	Regulations for Different GE Categories		
		SDN-1	SDN-2	SDN-3
Argentina	Non-ratified	Non-GMO	Cas-by-case decision	Non-GMO if no transgene
Australia	Non-ratified	Non-GMO	GMO	Not clear
Brazil	Ratified	Non-GMO (with previous consultation)	Cas-by-case decision	Non-GMO if no transgene
Canada	Non-ratified	Case-by-case (based upon novelty)		
Chile	Non-ratified	Non-GMO (with previous consultation)	Non-GMO	Non-GMO if no transgene
China	Ratified	Under review with new legislations		
Colombia	Ratified	Non-GMO (with previous consultation)	Non-GMO (Case-by-case decision)	Non-GMO if no transgene
European Union	Ratified	GMO		
Guatemala El Salvador	Ratified	No clear regulations		
Honduras	Ratified	Case-by-case non-GMO		Non-GMO if no transgene
India	Ratified	Currently unclear (under discussion)		
Israel	Non-ratified	Non-GMO		Non-GMO if no transgene
Japan	Ratified	Non-GMO if no extracellular footprints were integrated into the organism genome		GMO (if extracellular footprints remain)
New Zealand	Ratified	GMO		
Norway	Ratified	Currently unclear (proposal under review)		
Paraguay	Ratified	Unclear (may vary Case-by-case)		
Russian Federation	Non-ratified	Unclear due to new expected policies		
South Africa	Non-ratified	Currently unclear (under discussion)		
Switzerland	Ratified	Currently unclear (under discussion)		
United States	Non-ratified	Case-by-case (USDA); clarification under discussion (FDA)		

## Data Availability

Not applicable.
